# Gut microbiome taxonomic and functional capacity alterations in CHD and HFpEF

**DOI:** 10.3389/fmicb.2026.1875692

**Published:** 2026-10-13

**Authors:** Ainur Tauekelova, Nurlubek Katkenov, Samat Kozhakhmetov, Zharkyn Jarmukhanov, Elizaveta Vinogradova, Ayan Abdrakhmanov, Aliya Sailybayeva, Makhabbat Bekbossynova, Almagul Kushugulova

**Affiliations:** 1 Heart Center (Corporate Fund “University Medical Center”), Astana, Kazakhstan; 2 Laboratory of Microbiome, Center for Life Sciences, National Laboratory Astana, Nazarbayev University, Astana, Kazakhstan; 3 Heart Rhythm Scientific Research Institute, Astana Medical University, Astan, Kazakhstan

**Keywords:** coronary heart disease, gut microbiome, heart failure with preserved ejection fraction, microbiome functional profiling, shotgun metagenomics

## Abstract

The gut microbiome is known to modulate metabolic health, yet its role in coronary heart disease (CHD) and heart failure with preserved ejection fraction (HFpEF) remains unclear. In the present study, we profiled the gut microbiome of 234 participants using shotgun metagenomic sequencing across controls (*n* = 43), individuals with low-risk atherosclerosis (LRAS, *n* = 23), and patients with CHD + HFpEF (*n* = 168). Species richness was significantly increased in CHD + HFpEF, with no disruption in taxonomic evenness. LRAS did not diverge significantly in alpha metrics. In contrast, beta diversity analysis demonstrated significant divergence of both LRAS and CHD + HFpEF taxonomic structure from controls, with the greatest divergence in CHD + HFpEF, particularly in older patients. Notably, all differences were attenuated when comparing obese controls and disease groups. However, significant heterogeneity in multivariate dispersion was observed, and a contribution of differences in variation cannot be excluded. Further differential analysis, adjusting for age, BMI, sex, and glucose and lipid profiles, revealed significant associations between specific taxa and CHD + HFpEF, including depletion of the saccharolytic fermenter *Bifidobacterium* and enrichment of taxa linked to proteolytic or inflammatory metabolism (notably *Prevotella* and *Bilophila*), regardless of age and BMI. Subsequent analysis of inferred functional features indicated increased potential for L-citrulline, L-glutamate, L-lysine, and pyrimidine metabolism in CHD + HFpEF. Collectively, the present analysis suggests that CHD + HFpEF is associated with distinct compositional and functional shifts in the gut microbiome. Further longitudinal and interventional research is required to determine which of these microbial features contribute to disease progression and to better characterize underlying mechanisms.

## 1 Introduction

A growing body of recent evidence suggests that the gut microbiome plays a pivotal and previously underappreciated role in modulating cardiovascular risk. Increasing evidence indicates that intestinal microbial communities influence host metabolic homeostasis, systemic inflammation, endothelial function, and atherogenesis through complex host–microbe interactions, collectively contributing to the development and progression of cardiovascular disease (CVD) and supporting the concept of a bidirectional gut–heart axis (Tang et al., 2019; Trøseid et al., 2020; Kondapalli et al., 2025).

Indeed, the gut microbiota influences cardiovascular health through a multitude of mechanisms, including the modulation of systemic inflammation, lipid metabolism, and vascular ageing, with recent evidence highlighting both detrimental metabolites like TMAO and imidazole propionate, as well as protective microbial-derived compounds (Luqman et al., 2024; Snelson et al., 2025; Yu et al., 2025; Fusco et al., 2026).

Microbiota-derived metabolites constitute key mechanistic mediators of gut–heart crosstalk. Trimethylamine-N-oxide (TMAO), produced from dietary choline and carnitine via microbial transformation and hepatic oxidation, has been associated with incident cardiovascular events and adverse outcomes in heart failure cohorts (Wang et al., 2011; Tang et al., 2013; Haghikia et al., 2018). Conversely, short-chain fatty acids (SCFAs), generated by microbial fermentation of dietary fiber, exert anti-inflammatory effects and support intestinal barrier integrity. They also modulate vascular tone and myocardial metabolism through receptor-dependent signaling pathways (Koh et al., 2016; Xu et al., 2022). These complementary lines of evidence indicate that changes in microbial metabolic activity rather than taxonomic shifts alone can have direct consequences for cardiometabolic risk (Chulenbayeva et al., 2025).

Among cardiovascular syndromes potentially influenced by microbiome-derived mechanisms, heart failure with preserved ejection fraction (HFpEF) is of particular relevance. HFpEF is a heterogeneous clinical syndrome characterized by systemic inflammation, endothelial dysfunction, metabolic disturbances, and adverse myocardial remodeling (Borlaug and Paulus, 2011; Shah et al., 2016). Importantly, HFpEF’s strong epidemiologic links with aging, obesity, hypertension, and metabolic comorbidity overlap with factors known to remodel gut microbial communities, suggesting plausible mechanistic connections between host phenotype, microbial ecology, and disease expression (Beale et al., 2021; Huang et al., 2024).

Aging and adiposity deserve separate consideration as dominant modifiers of the gut microbiome. Older adults typically show reduced microbial diversity, loss of butyrate-producing taxa, greater inter-individual variability, and a pro-inflammatory milieu that can compromise barrier function and promote systemic inflammation. Similarly, obesity is associated with lower diversity, altered fermentative profiles, increased intestinal permeability, and metabolic endotoxemia, all of which may amplify cardiometabolic stress and confound disease-specific microbial signatures (O’Toole and Jeffery, 2015; Marzullo et al., 2020; Ghosh et al., 2022). Because these factors independently reprogram gut ecology, failure to account for age and obesity can obscure microbial signals that are specifically linked to cardiac pathology.

Despite growing interest in the gut–heart axis, most microbiome studies in heart failure have focused on systolic dysfunction or mixed populations and have relied predominantly on 16S rRNA sequencing without comprehensive functional characterization. HFpEF-specific investigations remain limited, particularly studies integrating high-resolution shotgun metagenomic profiling with pathway-level functional analysis. Moreover, few prior studies have systematically stratified participants by age and obesity status, despite the well-established impact of these factors on gut microbial composition and metabolic output. As a result, it remains unclear whether previously reported microbial alterations reflect disease-specific signatures or are driven by aging- and adiposity-related confounding. Addressing this gap is essential to disentangle HFpEF-associated microbial remodeling from background ecological shifts related to host phenotype. Accordingly, we aimed to characterize taxonomic and functional alterations in the gut microbiome in HFpEF using shotgun metagenomic sequencing and to evaluate their variation across age and obesity strata.

## 2 Materials and methods

### 2.1 Study population

This cross-sectional study included 234 adult participants: 43 healthy controls, 23 individuals with low atherosclerotic risk (LRAS), and 168 patients with coronary heart disease and heart failure with preserved ejection fraction (CHD + HFpEF). Participants were recruited at the Heart Center of the University Medical Center (Nazarbayev University, Astana, Kazakhstan) between March and December 2024. The study was conducted in accordance with the Declaration of Helsinki and approved by the Ethics Committee of the National Laboratory Astana, Nazarbayev University (protocol No. 05-2023, approved 21 November 2023).

Participants in the LRAS group were identified through cardiovascular screening as individuals with a low estimated cardiovascular risk. The control group comprised healthy volunteers with no cardiovascular risk factors or disease, based on clinical, laboratory, and instrumental evaluation. CHD + HFpEF patients were recruited from those undergoing cardiology evaluation and diagnosed per established criteria. Cardiovascular risk was classified using the European SCORE2 system: low risk defined as <2.5% for age <50 years, <5% for age 50–69, and <7.5% for age ≥70, in line with national dyslipidemia guidelines (SCORE2 working group and ESC Cardiovascular risk collaboration et al., 2021). HFpEF was diagnosed according to the 2021 European Society of Cardiology (ESC) guidelines, defined as the presence of heart failure symptoms and signs with preserved left ventricular ejection fraction (LVEF ≥50%) accompanied by evidence of structural heart disease and/or elevated natriuretic peptides (McDonagh et al., 2021). Patients were enrolled on the basis of an HFpEF diagnosis, with coronary heart disease recorded as a coexisting condition rather than as a separate inclusion criterion; the group designation CHD + HFpEF is therefore used throughout to denote this clinically defined cardiology cohort. All patients were recruited and evaluated within the same cardiology service. All participants underwent clinical evaluation, laboratory testing, and cardiovascular assessment, including measurement of ejection fraction (EF), total cholesterol (TC), glycated hemoglobin (HbA1c), C-reactive protein (CRP), high-density lipoprotein (HDL), low-density lipoprotein (LDL), triglycerides (TG), creatinine (CREAT), estimated glomerular filtration rate (eGFR), N-terminal pro-brain natriuretic peptide (NT-proBNP), and interleukin-6 (IL-6). However, NT-proBNP was not measured in the control. Data on arterial hypertension were collected.

Exclusion criteria applied to all study groups included the use of medications known to influence gut microbiota composition (including antibiotics, probiotics, or antiviral agents) within 3 months prior to enrollment, the presence of inflammatory or autoimmune disorders, significant chronic systemic diseases, immunodeficiency, gastrointestinal surgery or acute gastrointestinal infection within the preceding 6 months, pregnancy or lactation, and inability to provide informed consent. The control group additionally excluded individuals with any history of cardiovascular disease, family history of premature cardiovascular disease, or abnormal findings on clinical examination or laboratory testing.

### 2.2 Fecal sample collection

Fecal samples were collected from all participants within 48 h of study enrollment using DNA/RNA Shield™ Fecal Collection Tubes (Cat. No. R1101, Zymo Research, Irvine, CA, USA), following the manufacturer’s instructions. Participants received detailed written instructions for standardized sample collection. Samples were transported to the Microbiome Laboratory within 24 h of collection and immediately stored at −80 °C until further processing for metagenomic analysis.

### 2.3 DNA extraction and shotgun metagenomic sequencing

Microbial DNA was extracted from fecal samples using the ZymoBIOMICS DNA Miniprep Kit (Cat. No. D4301, Zymo Research, Irvine, CA, USA) according to the manufacturer’s standard protocol. The kit utilizes bead beating with high-density BashingBeads for efficient mechanical lysis, ensuring uniform disruption of resilient microbial cells, including Gram-positive bacteria and fungi; a high-speed cell disruptor was used to enhance homogenization. Sterile water was included as a negative extraction control. DNA quantity and quality were assessed using complementary approaches, including NanoDrop 2000 spectrophotometry (Thermo Fisher Scientific, USA), Qubit 3.0 fluorometric quantification (Invitrogen, USA), and agarose gel electrophoresis to evaluate DNA integrity. Shotgun metagenomic sequencing libraries were prepared and sequenced at Novogene (Beijing, China) on an Illumina NovaSeq 6,000 platform (Illumina, San Diego, CA, USA) using paired-end sequencing following standard Illumina protocols, with each sample generating an average of 6 Gb of raw sequencing data.

### 2.4 Bioinformatic processing and functional profiling

Data preprocessing was conducted using KneadData (v0.12.0), which removes contaminating sequences such as host DNA to improve data quality. Taxonomic profiling of microbial communities was performed with MetaPhlAn 4 using the mpa_vJan21_CHOCOPhlAnSGB202103 reference database, which identifies microbial taxa based on clade-specific marker genes. Functional profiling of microbial metabolic pathways was conducted using HUMAnN 3, with annotation against the UniRef90 database, enabling reconstruction of microbial metabolic pathways and functional potential within the gut microbiome. Default parameters were applied throughout the pipeline to maintain standardization and ensure compatibility across analytical stages.

### 2.5 Statistical analysis

Demographic, functional, and biochemical data on research participants in groups were compared using either the Kruskal-Wallis, one-way ANOVA, Mann–Whitney *U* rank test, or an independent *t*-test, depending on the data distribution. Normality was assessed using the Shapiro–Wilk test, and equality of variance was assessed using Levene’s test. Countable data were assessed using the chi-square or Fisher’s exact test. No adjustment for multiple comparisons was applied for the baseline descriptive analysis of participant parameters.

Alpha diversity was evaluated at the species level using Observed richness and Pielou’s evenness indices on default-output MetaPhlAn relative abundance data. Three comparisons were made for non-obese and obese groups: (1) CON vs. LRAS, (2) CON vs. CHD + HFpEF (age under 60), (3) CON vs. CHD + HFpEF (age over 60). Comparisons were carried out using the Mann–Whitney *U* test, and comparisons were adjusted using the Benjamini–Hochberg False Discovery Rate (FDR-BH) procedure. Differences were considered significant at *q* < 0.05.

Beta diversity was assessed using Bray–Curtis dissimilarity at the species level on Hellinger-transformed relative abundance data. Statistical differences in community composition between groups were evaluated using analysis of similarity (ANOSIM) with 999 permutations. Adonis2/PERMANOVA analysis (adjusting for age, BMI, sex, glucose and lipid profiles, inflammation status, and estimated kidney function) with a by-margin option was performed to analyze covariate associations with microbiome structure. ANOSIM and adonis2/PERMANOVA results were adjusted using the FDR-BH procedure, and associations were considered significant at *q* < 0.05. PERMDISP analysis with 999 permutations was used to investigate multivariate dispersion. Patterns were visualized using principal coordinates analysis (PCoA).

Differential analysis of taxonomic features was performed using MaAsLin3 v1.2.0 on relative normalized data, adjusting for age, BMI, sex, and glucose and lipid profiles. Log transformation was applied. Only features with a prevalence of at least 50% in the dataset were analyzed. Associations were considered significant at *q* < 0.1 (FDR-BH). Both abundance and prevalence models were considered. Differential analysis of inferred functional features was conducted subsequently, using the abundance model only; changes were considered significant at *q* < 0.05 (FDR-BH). The control group was set as the reference group.

Data processing, formal analyses, diversity analyses, and visualization were performed using pandas v3.0.1, NumPy v1.26.4, SciPy v1.11.4, statsmodels v0.14.6, scikit-bio v0.6.3, Matplotlib v3.10.8, and seaborn v0.13.2 in Python v3.12.2. Adonis2/PERMANOVA analysis using vegan v2.7.3 and differential analysis using MaAsLin3 v1.2.0 were conducted in R v4.5.3.

## 3 Results

### 3.1 Demographic and clinical characteristics of the study population

Demographic and clinical characteristics of the study population are summarized in Tables 1, 2. A total of 234 participants were enrolled in the study, of whom 138 were non-obese and 96 had a BMI ≥ 30 kg/m^2^. Given that both age and BMI are prominent expected contributors to vascular health, we subdivided and compared groups by obesity status and age. Figure 1 demonstrates a study population flow diagram.

**Table 1:** Demographic and clinical characteristics of the study population in non-obese (NO, BMI < 30 kg/m^2^) control (CON), low-risk atherosclerosis (LRAS), and coronary heart disease and heart failure with preserved ejection fraction (CHD + HFpEF) participants, with the CHD + HFpEF group stratified by age into <60 years and ≥60 years.

Variable	CON, NO, *n* = 34	LRAS, NO, *n* = 19	CHD + HFpEF (<60 years), NO, *n* = 35	*p*-value^¶^	CHD + HFpEF (≥60 years), NO, *n* = 50
Age, years	52.50 (45.25; 60.75)	49.00 (43.00; 55.5)	51.00 (45.50; 57.0)	*p* = 0.22^a^; *p* < 0.0001^b^	67.50 (63.25; 73.50)
BMI, kg/m^2^	23.60 (21.12; 26.98)	24.60 (23.02; 26.65)	26.00 (24.00; 27.50)	*p* < 0.01^a^; *p* = 0.78^b^	26.00 (25.00; 28.00)
Sex, male	47.06%	42.11%	51.43%	*p* = 0.17^d^; *p* = 0.52^e^	56.00%
EF, %	59.00 (56.00; 62.00)	63.00 (58.50; 64.50)	56.00 (53.00; 59.00)	*p* < 0.01^a^; *p* = 0.88^c^	56.50 (53.00; 59.00)
NT-proBNP, pg/mL	*NA*	77.94 (54.09; 153.2)	173.06 (65.26; 350.97)	*p* = 0.16^b^; *p* = 0.39^b^	172.27 (105.41; 343.23)
HbA1c, %	5.60 (5.31; 5.80)	5.10 (4.70; 5.55)	5.66 (5.40; 6.08)	*p* < 0.01^a^; *p* = 0.47^b^	5.80 (5.42; 6.24)
IL-6, pg/mL	4.28 (2.95; 5.41)	3.14 (2.24; 4.36)	5.74 (4.3; 8.62)	*p* < 0.001^a^; *p* = 0.11^b^	4.80 (3.62; 7.05)
CRP, mg/dL	0.12 (0.04; 0.21)	0.10 (0.04; 0.18)	0.16 (0.10; 0.52)	*p* = 0.06^a^; *p* = 0.16^b^	0.14 (0.08; 0.24)
HDL, mg/dL	58.16 (49.15; 65.17)	61.00 (53.74; 69.25)	46.13 (40.90; 64.70)	*p* = 0.11^a^; *p* = 0.07^b^	55.84 (48.67; 61.78)
LDL, mg/dL	105.88 (92.33; 119.42)	120.48 (111.50; 134.28)	125.63 (107.06; 146.80)	*p* < 0.01^a^; *p* = 0.42^b^	128.84 (105.13; 166.48)
TC, mg/dL	181.32 (165.35; 196.04)	186.9 (176.74; 205.03)	193.41 (178.55; 219.58)	*p* = 0.11^a^; *p* = 0.44^b^	205.88 (174.54; 242.72)
TG, mg/dL	75.08 (63.70; 129.48)	86.40 (52.70; 105.48)	110.11 (87.18; 184.17)	*p* < 0.01^a^; *p* = 0.89^b^	122.65 (92.84; 148.28)
CREAT, mg/dL	0.84 (0.79; 0.91)	0.83 (0.77; 0.93)	0.78 (0.67; 0.92)	*p* = 0.47^a^; *p* = 0.66^b^	0.86 (0.70; 0.91)
eGFR, mL/min/m^2^	97.72 (89.93; 102.78)	96.3 (89.2; 110.35)	103.12 (94.56; 107.4)	*p* = 0.30^a^; *p* < 0.001^b^	94.45 (81.36; 97.28)

^¶^Comparing CON, LRAS and CHD + HFpEF (<60 years); CHD + HFpEF (<60 years) and CHD + HFpEF (≥60 years).

^a^Kruskal-Wallis *H*-test; ^b^Mann-Whitney *U* rank test; ^c^Ind. *T*-test; ^d^Chi2; ^e^Fisher’s exact.

Data are presented as median and interquartile range (IQR) or *n* (%), as appropriate.

**Table 2:** Demographic and clinical characteristics of the study population in non-obese (OB, BMI ≥ 30 kg/m^2^) control (CON), low-risk atherosclerosis (LRAS), and coronary heart disease and heart failure with preserved ejection fraction (CHD + HFpEF) participants, with the CHD + HFpEF group stratified by age into <60 years and ≥60 years.

Variable	CON, OB, *n* = 9	LRAS, OB, *n* = 4	CHD + HFpEF (<60 years), OB, *n* = 51	*p*-value^¶^	CHD + HFpEF (≥60 years), OB, *n* = 32
Age, years	52.00 (48.00; 54.00)	51.50 (50.75; 53.50)	53.50 (50.75; 57.00)	*p* = 0.61^a^; *p* < 0.0001^b^	67.00 (63.00; 69.00)
BMI, kg/m^2^	31.00 (30.00; 32.00)	34.26 (33.80; 35.39)	33.00 (32.00; 36.00)	*p* = 0.06^a^; *p* = 0.29^b^	32.00 (31.00; 35.00)
Sex, male	77.78%	25.00%	59.38%	*p* = 0.20^d^; *p* = 0.38^e^	50.98%
EF, %	58.00 (57.00; 64.00)	63.50 (62.75; 64.25)	56.50 (54.00; 61.00)	*p* < 0.05^f^; *p* = 0.62^c^	56.00 (52.50; 59.00)
NT-proBNP, pg/mL	*NA*	132.01 (89.00; 201.13)	91.57 (36.65; 212.62)	*p* = 0.46^b^; *p* < 0.01^b^	159.17 (95.78; 319.78)
HbA1c, %	5.58 (5.53; 5.79)	5.60 (5.35; 5.82)	6.10 (5.78; 6.51)	*p* < 0.01^a^; *p* = 0.96^b^	6.08 (5.73; 6.64)
IL-6, pg/mL	4.42 (3.77; 5.24)	4.56 (3.91; 5.11)	4.49 (3.66; 7.5)	*p* = 0.91^a^; *p* = 0.81^b^	4.86 (3.37; 8.62)
CRP, mg/dL	0.16 (0.10; 0.63)	0.30 (0.13; 0.51)	0.27 (0.21; 0.51)	*p* = 0.74^a^; *p* = 0.13^b^	0.38 (0.22; 0.66)
HDL, mg/dL	46.6 (38.52; 48.22)	46.79 42.11; 50.70)	45.78 (42.06; 53.15)	*p* = 0.94^a^; *p* = 0.61^b^	46.76 (40.53; 56.24)
LDL, mg/dL	143.98 (113.0; 153.90)	154.37 (139.15; 166.10)	131.66 (110.40; 159.12)	*p* = 0.57^f^; *p* = 0.38^c^	124.72 (79.10; 149.08)
TC, mg/dL	200.69 (177.14; 219.70)	220.61 (202.53; 231.12)	194.73 (164.38; 225.57)	*p* = 0.64^f^; *p* = 0.73^c^	196.19 (155.8; 223.32)
TG, mg/dL	141.03 (113.82; 185.79)	158.62 (145.14; 168.12)	137.70 (102.10; 208.92)	*p* = 0.93^a^; *p* = 0.73^b^	138.41 (104.56; 186.32)
CREAT, mg/dL	0.88 (0.79; 0.93)	0.70 (0.61; 0.89)	0.81 (0.74; 0.90)	*p* = 0.56^b^; *p* = 0.42^b^	0.86 (0.72; 1.03)
eGFR, mL/min/m^2^	102.27 (99.00; 106.02)	101.30 (83.28; 114.50)	99.68 (90.92; 105.78)	*p* = 0.91^a^; *p* < 0.01^b^	91.09 (72.50; 97.94)

^¶^Comparing CON, LRAS and CHD + HFpEF (<60 years); CHD + HFpEF (<60 years) and CHD + HFpEF (≥60 years).

^a^Kruskal-Wallis *H*-test; ^b^Mann-Whitney *U* rank test; ^c^Ind. *T*-test; ^d^Chi2; ^e^Fisher’s exact; ^f^one-way ANOVA.

Data are presented as median and interquartile range (IQR) or *n* (%), as appropriate.

**Figure 1:**
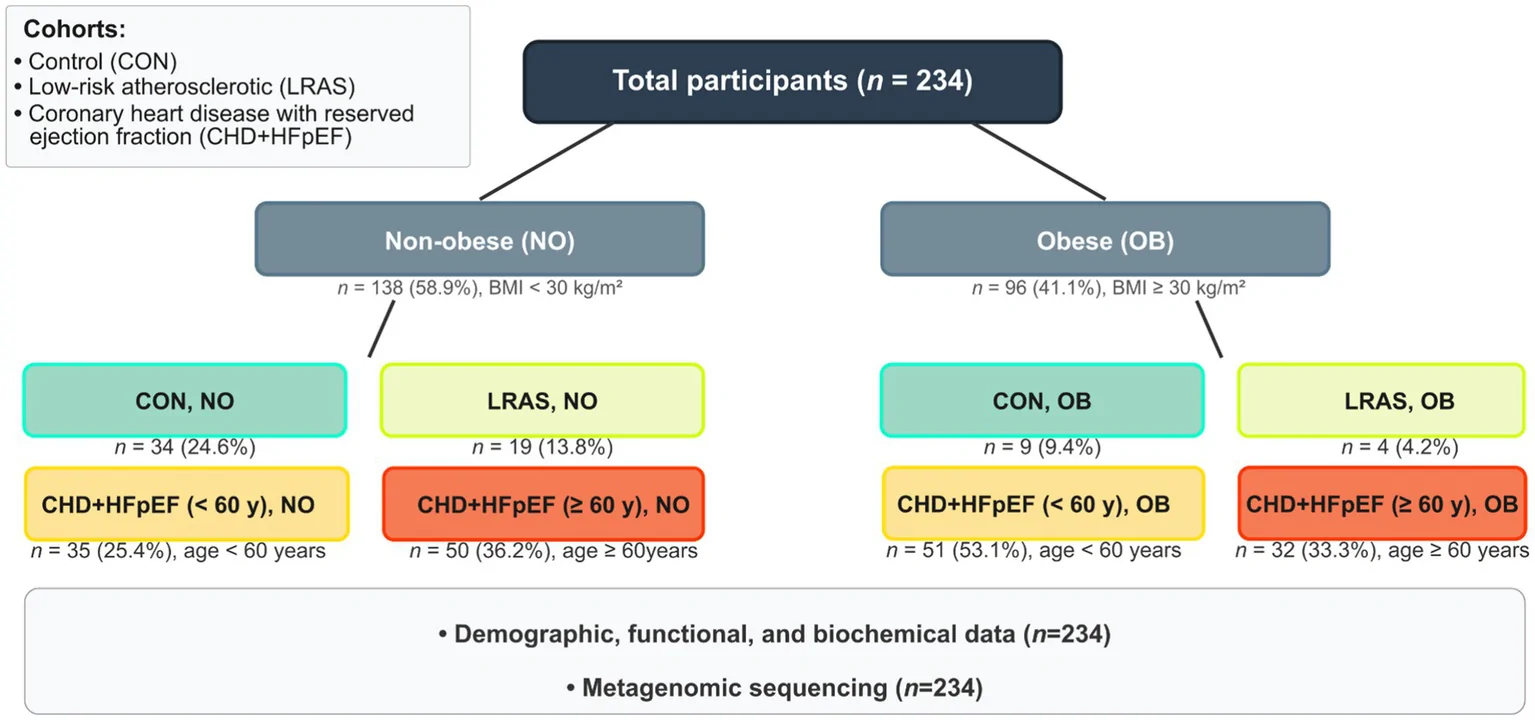
Study population diagram. Non-obese (NO, BMI < 30 kg/m^2^) and obese (OB, BMI ≥ 30 kg/m^2^) subgroups: control (CON), low-risk atherosclerotic (LRAS), and coronary heart disease and heart failure with preserved ejection fraction (CHD + HFpEF) participants. The CHD + HFpEF group is further stratified by age into <60 years and ≥60 years. BMI, Body Mass Index.

In non-obese groups (NO), the median age was 52.5 (interquartile range (IQR): 45.25; 60.75), 49.0 (43.00; 55.50), 51.00 (45.50; 57.00), and 67.50 (63.25; 73.50) for CON, LRAS, CHD + HFpEF (<60 years), and CHD + HFpEF (≥60 years), respectively. CON, LRAS, and CHD + HFpEF (<60 years) did not differ by age (*p* = 0.22). BMI was strictly below 30 kg/m^2^ in all participants, albeit with some variation between the groups (*p* < 0.01). Sex distribution was comparable across groups (*p* > 0.05). Ejection fraction varied between groups but remained above 50% in all participants. CRP levels did not differ significantly between groups (*p* > 0.05). LDL was higher in the LRAS and CHD + HFpEF groups compared to the CON, with median values of 105.88 (92.33; 119.42), 120.48 (111.50; 134.28), 125.63 (107.06; 146.80), and 128.84 (105.13; 166.48), respectively (Table 1).

In obese groups (OB), the median age was 52.00 (48.00; 54.00), 51.50 (50.75; 53.50), 53.50 (50.75; 57.00), and 67.00 (63.00; 69.00) for CON, LRAS, CHD + HFpEF (<60 years), and CHD + HFpEF (≥60 years), respectively. CON, LRAS, and CHD + HFpEF (<60 years) did not differ by age (*p* = 0.61). BMI was strictly above 30 kg/m^2^ in all participants, albeit with some variation between groups (*p* = 0.06). Sex distribution was comparable across groups (*p* > 0.05). Ejection fraction varied between groups but remained above 50% in all participants. CRP levels did not differ significantly between groups (*p* > 0.05). LDL was higher in the LRAS group compared to controls, with median values of 143.98 (113.00; 153.90), 154.37 (139.15; 166.10), 131.66 (110.40; 159.12), and 124.72 (79.10; 149.08), respectively (Table 2).

In summary, the study cohort comprised four distinct non-obese groups (*n* = 138) and four obese groups (*n* = 96), with age stratification applied exclusively to the CHD + HFpEF participants. Across both BMI strata, the control, LRAS, and CHD + HFpEF (<60 years) groups were matched for age, indicating that any differences observed in the main analyses will be unlikely to be driven by age disparities among these subgroups. As expected, the older CHD + HFpEF (≥60 years) subgroups were significantly older than their younger counterparts (*p* < 0.0001 for both non-obese and obese), justifying the age-based stratification. Sex distribution was comparable across all groups, while BMI, ejection fraction, and lipid profiles showed expected variations consistent with the group definitions. Diabetes mellitus was present in 58/168 (34.5%) of CHD + HFpEF patients. Additional echocardiographic characterization was available for a subset of the CHD + HFpEF group: the medial E/e′ ratio was 8.25 (IQR 6.44; 10.40), the lateral E/e′ ratio was 6.36 (IQR 4.49; 7.20), left atrial volume index was 30.90 (IQR 25.16; 39.43) mL/m^2^, and left ventricular mass, calculated using the Devereux formula, was 157.10 (IQR 127.10; 189.20) g. These parameters were not routinely assessed in the LRAS group, which was identified through cardiovascular risk screening rather than comprehensive cardiology evaluation.

### 3.2 Biodiversity analysis

#### 3.2.1 Alpha diversity: species richness and evenness

Alpha diversity was assessed using Observed richness (measuring species count) and Pielou’s evenness index (measuring abundance distribution) (Figures 2A,B).

**Figure 2:**
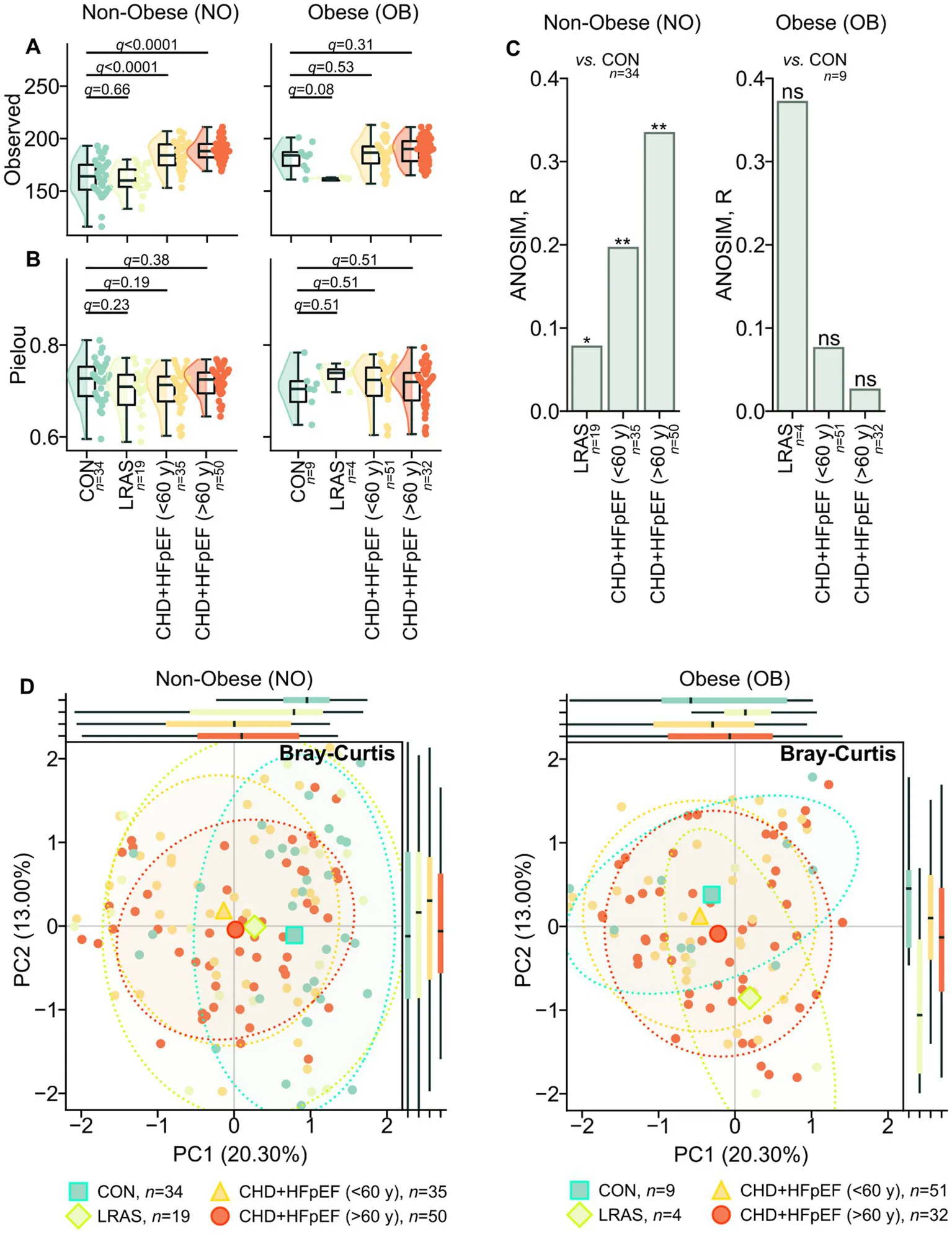
Alpha and beta diversity of the gut microbiome across non-obese (NO) and obese (OB) strata in the control (CON), low-risk atherosclerosis (LRAS), coronary heart disease and heart failure with preserved ejection fraction under 60 years old (CHD + HFpEF <60 years), and over 60 years old (CHD + HFpEF ≥60 years) groups. **(A)** Observed richness, **(B)** Pielou’s evenness. Mann–Whitney *U* test, FDR-BH. **(C)** Panels show ANOSIM R statistics comparing each group with controls, FDR-BH. **(D)** Principal coordinates analysis (PCoA) based on Bray–Curtis dissimilarity. ^*^q < 0.05, ^**^q < 0.01.

In the non-obese stratum, Observed richness was significantly higher in both CHD + HFpEF subgroups (younger <60 and older ≥60) compared to the control group (*q* < 0.001). No significant differences were observed between the control and LRAS groups. In the obese stratum, Observed richness did not differ significantly across any of the groups.

Pielou’s evenness index showed no significant differences between any groups in either BMI stratum (*q* > 0.05 for all comparisons), suggesting that the relative abundance distribution of the microbial community did not show prominent disruption across clinical phenotypes.

#### 3.2.2 Beta diversity: structural dissimilarity of microbial communities

Differences in microbiome composition structure were assessed using Bray–Curtis dissimilarity and visualized by principal coordinates analysis (PCoA) (Figures 2C,D).

Similarly to alpha diversity among non-obese participants, microbial community composition differed across disease groups (Figure 2C). ANOSIM analysis showed differences in microbial community composition relative to controls, with ANOSIM R values of 0.08 (*q* < 0.05) for the LRAS group, 0.20 (*q* < 0.01) for CHD + HFpEF patients younger than 60 years, and 0.33 (*q* < 0.01) for those older than 60 years. In obese participants, no difference from controls (*q* > 0.05) was observed. The first two principal coordinates explained 20.30 and 13.00% of the total variance, respectively (Figure 2D).

We further performed an adonis2/PERMANOVA analysis (adjusting for age, BMI, sex, glucose and lipid profile, inflammation status, and estimated kidney function) using a by-margin option to analyze covariate associations with microbiome structure. Experimental grouping explained 3% of the variance (*R*^2^ = 0.03). In addition to experimental grouping, BMI, LDL, and estimated kidney function showed the strongest associations with microbiome structure (*R*^2^ ≥ 0.01), though all explained only a modest proportion of variance (Table 3).

**Table 3:** Adonis2/PERMANOVA analysis of gut microbiome structure at the species level across control (CON), low-risk atherosclerosis (LRAS), and coronary heart disease and heart failure with preserved ejection fraction (CHD + HFpEF) groups, adjusting for key demographic and biochemical parameters.

Statum	*R* ^2^	*F*	*Q*-value
Group, df = 2	0.03	4.41	*q* = 0.005
Age, df = 1	0.008	2.28	*q* = 0.026
Sex, df = 1	0.008	2.26	*q* = 0.030
BMI, df = 1	0.01	4.17	*q* = 0.005
HbA1c, df = 1	0.008	2.13	*q* = 0.030
LDL, df = 1	0.01	2.66	*q* = 0.017
HDL, df = 1	0.004	1.17	*q* = 0.254
CRP, df = 1	0.008	2.17	*q* = 0.030
IL6, df = 1	0.006	1.63	*q* = 0.087
eGFR, df = 1	0.01	2.65	*q* = 0.017

Hellinger-transformed data, by-margin option, FDR-BH.

Additionally, we performed PERMDISP analysis (Table 4), which indicated significant heterogeneity in multivariate dispersion between non-obese controls and CHD + HFpEF groups (*p* < 0.05). Thus, it cannot be excluded that beta-diversity differences were driven by differences in variation.

**Table 4:** PERMDISP results for beta diversity analysis, comparing corresponding controls (CON) to experimental groups: in the control low-risk atherosclerosis (LRAS), coronary heart disease and heart failure with preserved ejection fraction under 60 years old (CHD + HFpEF <60 years), and over 60 years old (CHD + HFpEF ≥60 years) across non-obese (NO) and obese (OB) strata.

Statum	Group	Test	Statistic	*p*-value
NO	LRAS	PERMDISP	*F* = 0.47	*p* = 0.508
	CHD + HFpEF (<60 years)	PERMDISP	*F* = 39.71	*p* = 0.001
	CHD + HFpEF (≥60 years)	PERMDISP	*F* = 55.36	*p* = 0.001
OB	LRAS	PERMDISP	*F* = 1.62	*p* = 0.273
	CHD + HFpEF (<60 years)	PERMDISP	*F* = 0.34	*p* = 0.616
	CHD + HFpEF (≥60 years)	PERMDISP	*F* = 0.02	*p* = 0.921

### 3.3 Gut microbiome composition

At the phylum level, the gut microbiome across all study groups was predominantly composed of Bacillota (formerly Firmicutes) and Bacteroidota (formerly Bacteroidetes), with smaller contributions from Pseudomonadota (formerly Proteobacteria), Actinomycetota (formerly Actinobacteria), and Verrucomicrobiota (formerly Verrucomicrobia). The overall gut microbiome structure at the phylum and genus levels based on relative abundance profiles is presented in Figures 3A,B.

**Figure 3:**
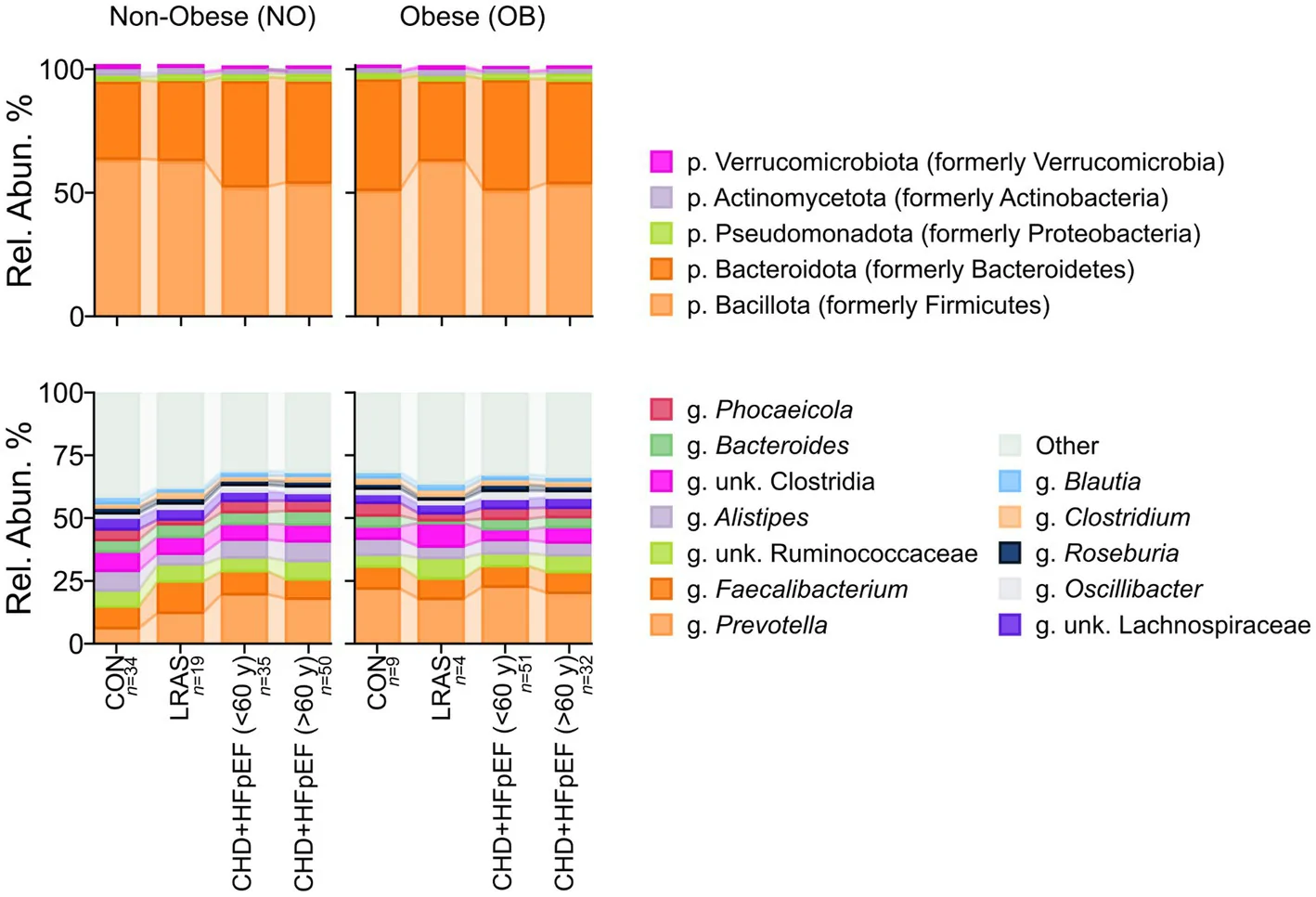
Relative abundance of gut microbiome taxa at the phylum and genus levels across non-obese (NO) and obese (OB) strata in the control (CON), low-risk atherosclerosis (LRAS), and coronary heart disease and heart failure with preserved ejection fraction groups, the latter stratified by age into <60 years (CHD + HFpEF <60 years) and ≥60 years (CHD + HFpEF ≥60 years). **(A)** Median abundance at the phylum level. **(B)** Median abundance at the genus level.

Across all study groups, the microbial community was dominated by *Prevotella* and *Faecalibacterium*, as well as unclassified (unk.) taxa belonging to the family Ruminococcaceae. Additional commonly detected taxa included *Alistipes*, unk. Clostridia, *Bacteroides*, *Phocaeicola*, unk. Lachnospiraceae, *Oscillibacter*, *Roseburia*, *Clostridium*, and *Blautia*.

### 3.4 Differential abundance analysis

#### 3.4.1 Differential abundance analysis of taxonomic features

We further performed an analysis of taxonomic features using MaAsLin 3 regression (adjusting for age, BMI, sex, glucose and lipid profile, inflammation status, and estimated kidney function), evaluating both prevalence and abundance.

At the genus level (Figure 4), CHD + HFpEF was associated with significantly higher *Prevotella* (phylum Bacteroidota) abundance and prevalence independent of age and BMI (*q* < 0.005) compared to controls. Similarly, *Adlercreutzia* (phylum Actinomycetota), *Bilophila* (phylum Pseudomonadota), *Duodenibacillus* (phylum Pseudomonadota), *Phascolarctobacterium* (phylum Bacillota), *Phocea* (phylum Bacillota), and *Lachnospira* (phylum *Bacillota*) were also associated with increased abundance and prevalence (*q* = 0.09), although the association was less strong. *Bifidobacterium* (phylum Actinomycetota) abundance and prevalence appeared to be depleted in CHD + HFpEF (*q* = 0.09).

**Figure 4:**
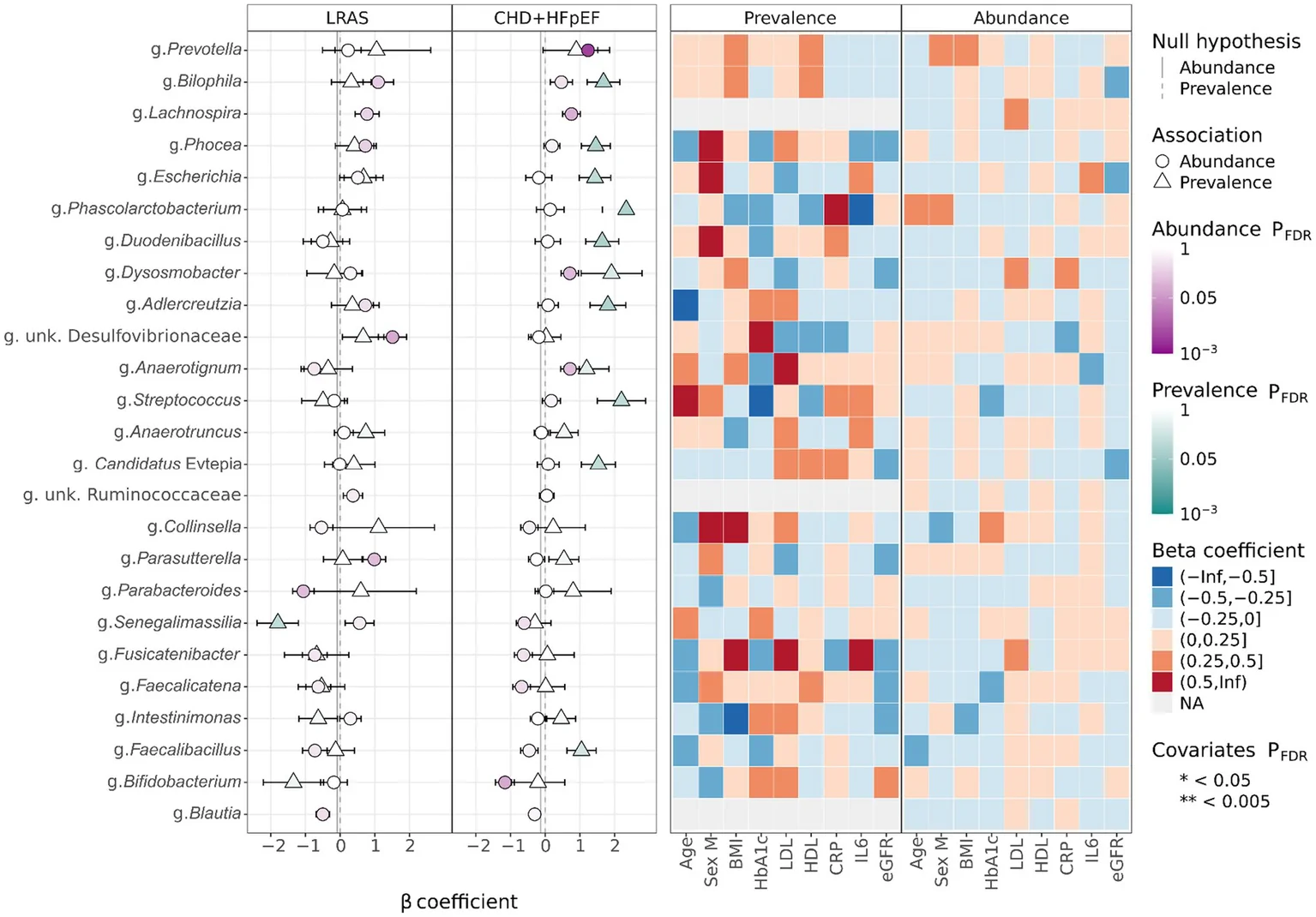
Differential analysis of gut microbiome taxonomic features at the genus level across control (CON), low-risk atherosclerosis (LRAS), and coronary heart disease and heart failure with preserved ejection fraction (CHD + HFpEF) groups, adjusting for key demographic and biochemical parameters. MaAsLin 3 regression analysis, log transform, FDR-BH.

In contrast, low-risk atherosclerosis was not associated with significant differences in this adjusted model compared to controls, with the exception of unk. Desulfovibrionaceae, which was associated with elevated abundance and prevalence (*q* = 0.09). Notably, *Bilophila* abundance was also elevated, although this did not remain significant after multiple-comparison correction (*p* = 0.009, *q* = 0.23).

Accordingly, in addition to genus-level profiling, CHD + HFpEF was associated with increased total abundance of Bacteroidota (*q* = 0.0004) and Pseudomonadota (*q* = 0.002) at the phylum level, whereas low-risk atherosclerosis was not associated with significant changes.

#### 3.4.2 Differential abundance analysis of inferred functional features

Subsequent analysis of inferred functional capacity indicated a difference in metabolic potential in CHD + HFpEF patients compared to controls. Analysis suggested a significant increase in the potential for L-citrulline, L-glutamate, L-lysine, and pyrimidine metabolism in CHD + HFpEF, and to some extent in LRAS (Figure 5).

**Figure 5:**
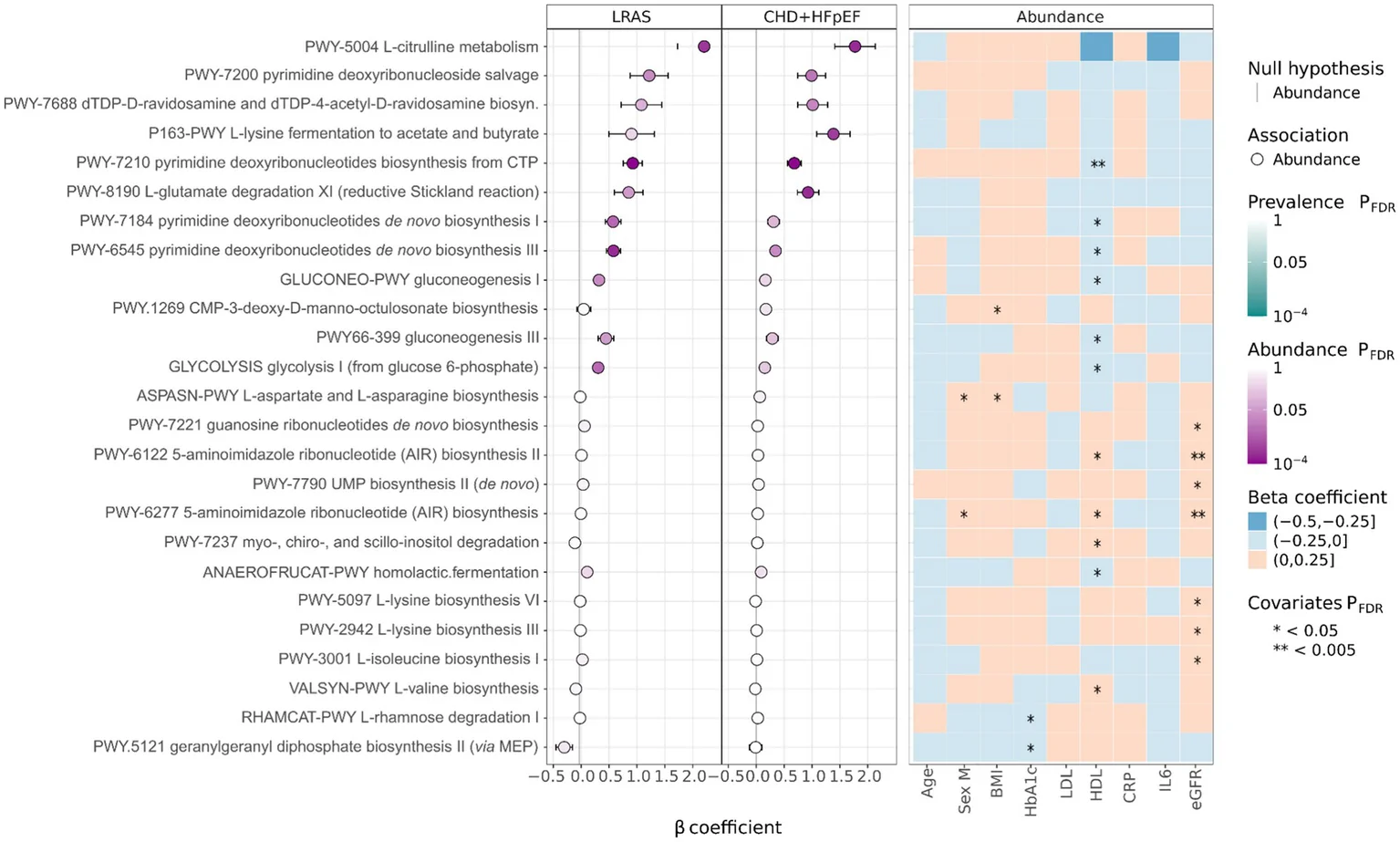
Differential analysis of gut microbiome inferred functional features across control (CON), low-risk atherosclerosis (LRAS), and coronary heart disease and heart failure with preserved ejection fraction (CHD + HFpEF) groups, adjusting for key demographic and biochemical parameters. MaAsLin 3 regression analysis, log transform, FDR-BH.

In particular, both LRAS and CHD + HFpEF groups were associated with increased capacity for (*q* < 0.05) L-citrulline metabolism (PWY-5004), pyrimidine deoxyribonucleotides *de novo* biosynthesis (PWY-6545), pyrimidine deoxyribonucleotides biosynthesis from CTP, pyrimidine deoxyribonucleoside salvage (PWY-7200), and L-glutamate degradation (PWY-7210).

At the same time, CHD + HFpEF was uniquely associated (*q* < 0.05) with altered capacity for L-lysine fermentation to acetate and butanoate (P163-PWY), and LRAS was uniquely associated with gluconeogenesis capacity (GLUCONEO-PWY and PWY66-399), glycolysis (GLYCOLYSIS and PWY-5484), and mixed acid fermentation (FERMENTATION-PWY).

## 4 Discussion

In the present study, we profiled the gut microbiome of 234 participants using shotgun metagenomic sequencing across controls, individuals with low-risk atherosclerosis (LRAS), and patients with coronary heart disease and heart failure with preserved ejection fraction (CHD + HFpEF). CHD + HFpEF was found to be distinct from controls, whereas LRAS was associated with less pronounced separation. Alpha diversity analysis suggested a significant increase in diversity in CHD + HFpEF, with no disruption in taxonomic evenness. LRAS was not associated with significant divergence in alpha metrics. Beta diversity analysis, by contrast, demonstrated significant divergence of both LRAS and CHD + HFpEF taxonomic structure from controls, although CHD + HFpEF demonstrated the greatest divergence, particularly in older CHD + HFpEF patients. Notably, all differences in diversity structure were attenuated when comparing obese controls and disease groups. Nonetheless, it should be noted that groups exhibited significant heterogeneity in multivariate dispersion; thus, it cannot be excluded that beta-diversity differences were driven by differences in variation.

Further differential analysis, adjusting for age, body mass index (BMI), sex, and glucose and lipid profiles, revealed a significant association between specific taxonomic features and CHD + HFpEF. CHD + HFpEF samples were associated with significant depletion of saccharolytic fermenter *Bifidobacterium*, and enrichment in taxa previously associated with proteolytic or inflammatory metabolism (notably *Prevotella* and *Bilophila*), regardless of age and BMI. LRAS, however, was associated with less divergence from controls. At a broader taxonomic level, these shifts correspond to a relative expansion of Bacteroidota and Pseudomonadota (formerly Proteobacteria) lineages, a pattern commonly associated with inflammatory and metabolically altered gut environments. Subsequent analysis of inferred functional capacity indicated a difference in metabolic potential in CHD + HFpEF patients compared to controls. Analysis suggested a significant increase in the potential for L-citrulline, L-glutamate, L-lysine, and pyrimidine metabolism in CHD + HFpEF, and to some extent in LRAS. Overall, these data suggest that CHD + HFpEF is associated with differences in gut microbiome taxonomic structure and metabolic potential, with LRAS displaying intermediate, less pronounced differences.

These results align with an expanding literature that positions the gut microbiome as a modulator of cardiometabolic physiology and cardiovascular risk (Trøseid et al., 2020; Witkowski et al., 2020). Prior clinical and experimental work implicates microbially derived metabolites and microbe–host interactions in blood pressure regulation, atherogenesis, and heart failure pathophysiology (Marques et al., 2018; Witkowski et al., 2020; Papadopoulos et al., 2022). Classic examples include microbiome-dependent generation of trimethylamine and its hepatic oxidation product TMAO, which have been linked epidemiologically and mechanistically to atherosclerotic risk and adverse cardiovascular outcomes (Wang et al., 2011; Tang et al., 2014; Li et al., 2017).

The progressive divergence of community composition from controls to LRAS to CHD + HFpEF indicates that detectable microbiome remodeling may occur early in cardiometabolic disease and intensify with disease progression. This gradient is consistent with studies linking gut dysbiosis to preclinical cardiometabolic traits, including hypertension and metabolic syndrome (Yang et al., 2015; Marques et al., 2018). These patterns indicate that the LRAS group occupies a position between controls and CHD + HFpEF along this gradient, but they do not establish causality: microbiome changes could be a cause, a consequence, or a co-evolving feature of cardiometabolic pathology.

Overall, given the depletion of saccharolytic fermenters and the enrichment of proteolytic or inflammatory metabolism-associated taxa observed in this study, potential mechanistic links between gut microbiome alterations and systemic inflammation in CHD + HFpEF may involve changes in intestinal barrier function and microbial metabolite production. These mechanisms may contribute to adverse cardiac remodeling, including myocardial fibrosis, cardiomyocyte hypertrophy, and extracellular matrix remodeling, and may ultimately influence the development and progression of heart failure with preserved ejection fraction, warranting further research.

Several limitations of the present study should be considered when interpreting the results. First, the present study’s cross-sectional design limits our ability to interpret the position of the LRAS group along the disease gradient and precludes any causal inference. Second, in this study we relied on inferred pathway abundances from metagenomic data rather than direct metabolite quantification. Thus, the inferred functional profiles may not reflect actual metabolite concentrations. These predictions require further validation and should be interpreted with caution. Third, although gut barrier dysfunction has been proposed as a potential contributor to systemic inflammation in heart failure, these parameters were not assessed in the present study. Integration of barrier markers and systemic inflammation panels with microbiome and metabolomics data is further required. Finally, several potential confounders that were not accounted for in the present study require explicit consideration. Diet and commonly used medications (for example, statins, PPIs, and ACEi/ARBs) are both potential modifiers of gut microbial composition (Vich Vila et al., 2020). However, diet and medications were not analysed in the current study. Potential residual confounding therefore cannot be excluded, warranting additional analysis.

## 5 Conclusion

In summary, the present analysis suggests that coronary heart disease and heart failure with preserved ejection fraction is associated with compositional and functional shifts in the gut microbiome. Further research is required to determine which of these microbial features contribute to disease progression and which are reflective of disease state. Critical next steps include longitudinal and interventional studies, as well as experiments with targeted metabolomics.

## Data Availability

The datasets presented in this study can be found in online repositories. The names of the repository/repositories and accession number(s) can be found at: https://www.ncbi.nlm.nih.gov/, PRJNA1274361.

## References

[ref1] Beale A. L. O’Donnell J. A. Nakai M. E. Nanayakkara S. Vizi D. Carter K. . (2021). The gut microbiome of heart failure with preserved ejection fraction. J. Am. Heart Assoc. 10:e020654. doi: 10.1161/JAHA.120.020654, 34212778 PMC8403331

[ref2] Borlaug B. A. Paulus W. J. (2011). Heart failure with preserved ejection fraction: pathophysiology, diagnosis, and treatment. Eur. Heart J. 32, 670–679. doi: 10.1093/eurheartj/ehq426, 21138935 PMC3056204

[ref3] Chulenbayeva L. Issilbayeva A. Sailybayeva A. Bekbossynova M. Kozhakhmetov S. Kushugulova A. (2025). Short-chain fatty acids and their metabolic interactions in heart failure. Biomedicine 13:343. doi: 10.3390/biomedicines13020343, 40002756 PMC11853371

[ref4] Fusco W. Adolph T. Cammarota G. Gasbarrini A. Ianiro G. Tilg H. (2026). Gut microbiota and atherosclerosis. Gut 75, 1067–1077. doi: 10.1136/gutjnl-2025-335610, 40645765

[ref5] Ghosh T. S. Shanahan F. O’Toole P. W. (2022). The gut microbiome as a modulator of healthy ageing. Nat. Rev. Gastroenterol. Hepatol. 19, 565–584. doi: 10.1038/s41575-022-00605-x, 35468952 PMC9035980

[ref6] Haghikia A. Li X. S. Liman T. G. Bledau N. Schmidt D. Zimmermann F. . (2018). Gut microbiota–dependent trimethylamine N-oxide predicts risk of cardiovascular events in patients with stroke and is related to proinflammatory monocytes. Arterioscler. Thromb. Vasc. Biol. 38, 2225–2235. doi: 10.1161/ATVBAHA.118.311023, 29976769 PMC6202215

[ref7] Huang J. Lin Y. Ding X. Lin S. Li X. Yan W. . (2024). Alteration of the gut microbiome in patients with heart failure: a systematic review and meta-analysis. Microb. Pathog. 192:106647. doi: 10.1016/j.micpath.2024.106647, 38788811

[ref8] Koh A. De Vadder F. Kovatcheva-Datchary P. Bäckhed F. (2016). From dietary fiber to host physiology: short-chain fatty acids as key bacterial metabolites. Cell 165, 1332–1345. doi: 10.1016/j.cell.2016.05.041, 27259147

[ref9] Kondapalli N. Katari V. Dalal K. K. Paruchuri S. Thodeti C. K. (2025). Microbiota in gut-heart axis: metabolites and mechanisms in cardiovascular disease. Compr. Physiol. 15:e70024. doi: 10.1002/cph4.70024, 40542540 PMC12181760

[ref10] Li X. S. Obeid S. Klingenberg R. Gencer B. Mach F. Räber L. . (2017). Gut microbiota-dependent trimethylamine N-oxide in acute coronary syndromes: a prognostic marker for incident cardiovascular events beyond traditional risk factors. Eur. Heart J. 38, 814–824. doi: 10.1093/eurheartj/ehw582, 28077467 PMC5837488

[ref11] Luqman A. Hassan A. Ullah M. Naseem S. Ullah M. Zhang L. . (2024). Role of the intestinal microbiome and its therapeutic intervention in cardiovascular disorder. Front. Immunol. 15:1321395. doi: 10.3389/fimmu.2024.1321395, 38343539 PMC10853344

[ref12] Marques F. Z. Mackay C. R. Kaye D. M. (2018). Beyond gut feelings: how the gut microbiota regulates blood pressure. Nat. Rev. Cardiol. 15, 20–32. doi: 10.1038/nrcardio.2017.120, 28836619

[ref13] Marzullo P. di Renzo L. Pugliese G. de Siena M. Barrea L. Muscogiuri G. . (2020). From obesity through gut microbiota to cardiovascular diseases: a dangerous journey. Int. J. Obes. Suppl. 10, 35–49. doi: 10.1038/s41367-020-0017-1, 32714511 PMC7371682

[ref9051] McDonagh T. A. Metra M. Adamo M. Gardner R. S. Baumbach A. Böhm M. (2021). ESC Scientific Document Group. 2021 ESC Guidelines for the diagnosis and treatment of acute and chronic heart failure. European Heart Journal. 42, 3599–3726. doi: 10.1093/eurheartj/ehab368, 34922348

[ref14] O’Toole P. W. Jeffery I. B. (2015). Gut microbiota and aging. Science 350, 1214–1215. doi: 10.1126/science.aac8469, 26785481

[ref15] Papadopoulos P. D. Tsigalou C. Valsamaki P. N. Konstantinidis T. G. Voidarou C. Bezirtzoglou E. (2022). The emerging role of the gut microbiome in cardiovascular disease: current knowledge and perspectives. Biomedicine 10:948. doi: 10.3390/biomedicines10050948, 35625685 PMC9139035

[ref16] Shah S. J. Kitzman D. W. Borlaug B. A. van Heerebeek L. Zile M. R. Kass D. A. . (2016). Phenotype-specific treatment of heart failure with preserved ejection fraction: a multiorgan roadmap. Circulation 134, 73–90. doi: 10.1161/CIRCULATIONAHA.116.021884, 27358439 PMC4930115

[ref17] Snelson M. Muralitharan R. Liu C. F. Markó L. Forslund S. K. Marques F. Z. . (2025). Gut-heart Axis: the role of gut microbiota and metabolites in heart failure. Circ. Res. 136, 1382–1406. doi: 10.1161/CIRCRESAHA.125.325516, 40403109 PMC12101525

[ref18] Tang W. H. W. Li D. Y. Hazen S. L. (2019). Dietary metabolism, the gut microbiome, and heart failure. Nat. Rev. Cardiol. 16, 137–154. doi: 10.1038/s41569-018-0108-7, 30410105 PMC6377322

[ref19] Tang W. H. W. Wang Z. Fan Y. Levison B. Hazen J. E. Donahue L. M. . (2014). Prognostic value of elevated levels of intestinal microbe-generated metabolite trimethylamine-N-oxide in patients with heart failure. J. Am. Coll. Cardiol. 64, 1908–1914. doi: 10.1016/j.jacc.2014.02.617, 25444145 PMC4254529

[ref20] Tang W. H. W. Wang Z. Levison B. S. Koeth R. A. Britt E. B. Fu X. . (2013). Intestinal microbial metabolism of phosphatidylcholine and cardiovascular risk. N. Engl. J. Med. 368, 1575–1584. doi: 10.1056/NEJMoa1109400, 23614584 PMC3701945

[ref21] Trøseid M. Andersen G. Ø. Broch K. Hov J. R. (2020). The gut microbiome in coronary artery disease and heart failure: current knowledge and future directions. EBioMedicine 52:102649. doi: 10.1016/j.ebiom.2020.102649, 32062353 PMC7016372

[ref22] Vich Vila A. Collij V. Sanna S. Sinha T. Imhann F. Bourgonje A. R. . (2020). Impact of commonly used drugs on the composition and metabolic function of the gut microbiota. Nat. Commun. 11:362. doi: 10.1038/s41467-019-14177-z, 31953381 PMC6969170

[ref23] Wang Z. Klipfell E. Bennett B. J. Koeth R. Levison B. S. DuGar B. . (2011). Gut flora metabolism of phosphatidylcholine promotes cardiovascular disease. Nature 472, 57–63. doi: 10.1038/nature09922, 21475195 PMC3086762

[ref24] Witkowski M. Weeks T. L. Hazen S. L. (2020). Gut microbiota and cardiovascular disease. Circ. Res. 127, 553–570. doi: 10.1161/CIRCRESAHA.120.316242, 32762536 PMC7416843

[ref25] Xu J. Moore B. N. Pluznick J. L. (2022). Short-chain fatty acid receptors and blood pressure regulation: council on hypertension mid-career award for research excellence 2021. Hypertension 79, 2127–2137. doi: 10.1161/HYPERTENSIONAHA.122.18558, 35912645 PMC9458621

[ref26] Yang T. Santisteban M. M. Rodriguez V. Li E. Ahmari N. Carvajal J. M. . (2015). Gut Dysbiosis is linked to hypertension. Hypertension 65, 1331–1340. doi: 10.1161/HYPERTENSIONAHA.115.05315, 25870193 PMC4433416

[ref27] Yu J. Yang Y. N. Chen W. Hu J. Jin Z. Wu C. . (2025). Role of gut microbiota and derived metabolites in cardiovascular diseases. iScience 28:113247. doi: 10.1016/j.isci.2025.113247, 40894914 PMC12392330

